# Synthesis and Evaluation of [^67^Ga]-AMD3100: A Novel Imaging Agent for Targeting the Chemokine Receptor CXCR4

**DOI:** 10.3797/scipharm.1305-18

**Published:** 2013-09-12

**Authors:** Ayuob Aghanejad, Amir R. Jalilian, Yousef Fazaeli, Behrouz Alirezapoor, Mehraban Pouladi, Davoud Beiki, Stephan Maus, Ali Khalaj

**Affiliations:** 1 Research Center for Nuclear Medicine, Tehran University of Medical Sciences, Tehran, Iran.; 2 Department of Nuclear Pharmacy, Faculty of Pharmacy, Tehran University of Medical Sciences, Tehran, Iran.; 3 Radiation Application Research School, Nuclear Science and Technology Research Institute, Tehran, 11365-3486, Iran.; 4 Clinic of Nuclear Medicine, University Medical Centre Mainz, Langenbeckstr. 1, D-55131 Mainz, Germany.

**Keywords:** Gallium-67, CXCR4, Biodistribution, SPECT, Breast carcinoma

## Abstract

In order to develop a possible C-X-C chemokine receptor type 4 (CXCR4) imaging agent for oncological scintigraphy, [^67^Ga]-labeled 1,1′-[1,4-Phenylene-bis(methylene)]bis(1,4,8,11-tetraazacyclotetradecane) ([^67^Ga]-AMD3100) was prepared by using [^67^Ga]GaCl_3_ and AMD-3100 for 2 h at 50 °C (radiochemical purity: >95% ITLC, >99% HPLC, specific activity: 1800–2000 TBq/mmol) in acetate buffer. The stability of the complex was checked in the presence of human serum (37 °C) and in the final formulation for four days. The biodistribution of the labeled compound in the vital organs of wild type Sprague-Dawley rats was determined and compared with that of the free Ga^3+^ cation up to 48 h. Considering the spleen as the target organ, the best target:non target ratios were obtained 48 h post-injection (spleen:blood ratio; 14.5 and spleen:muscle ratio; 88.4). Initial SPECT images and biodistribution results in the wild type rats matched each other and demonstrated rapid washout of the tracer from the urinary tract. SPECT images in human breast carcinoma-bearing mice demonstrated a detectable tumor uptake in 48 h post-injection.

## Introduction

The chemokine receptor subtype CXCR4 is an attractive target for cancer diagnosis and treatment as it is overexpressed in more than 70% of human solid tumors, including mammary cancer, prostate cancer, B-cell lymphoma, neuroblastoma, melanoma, cervical adenocarcinoma, and glioma [1]. Moreover, it is involved in three fundamental aspects of cancer: primary tumor growth, cancer cell migration, and establishment of metastatic sites. Many peptidic and nonpeptidic ligands with different modes of antagonistic activity have been developed against this receptor [2].

Recent studies confirmed the necessity of CXCR4 in breast cancer metastasis [3] and imaging studies demonstrated that CXCR4 is required to initiate the proliferation and/or promote survival of breast cancer cells *in vivo* and suggest that CXCR4 inhibitors, such as; 1,1′-[1,4-Phenylenebis(methylene)]bis(1,4,8,11-tetraazacyclotetradecane) (AMD3100; Plerixafor), can improve the treatment of patients with primary and metastatic breast cancers [4].

Previous studies have demonstrated that metals bound to the cyclam core increase the affinity of AMD3100 to the CXCR4 receptor, for instance, a copper complex affinity is increased by 6-fold [5]. Also, the binuclear ZnII, CuII, and NiII complexes of AMD3100 have shown to enhance the binding properties of AMD3100. Interestingly, the Zn(II)-AMD3100 complex (carrying an overall +4 charge), revealed a marginally higher specificity and reduced toxicity *in vitro* compared to the free ligand [6]. Also, it was shown that the increased binding affinity of the single-metal-ion-substituted AMD3100 is obtained through the enhanced interaction of one of the cyclam ring systems with the carboxylate group of the CXCR4 receptor and not binuclear complexes [5].

To image CXCR4 expression in tumors using positron emission tomography (PET) and single photon emission tomography (SPECT), various groups have utilized the ability of the cyclam function of the prototype CXCR4 inhibitor, AMD3100 to form strong complexes with radionuclides including Tc-99m [7]. Cu-64 [8] has also shown potential in human xenograft imaging using PET [9]. The interesting physical properties and availability of gallium-67 make it an interesting nuclide for radiopharmaceutical research [10].

The increasing trend in the production and use of PET radionuclides in nuclear medicine has offered new opportunities for researchers to focus on the production of new Ga-radiopharmaceuticals for feasibility studies using Ga-67 for their future PET gallium homologs.

**Fig. 1:**
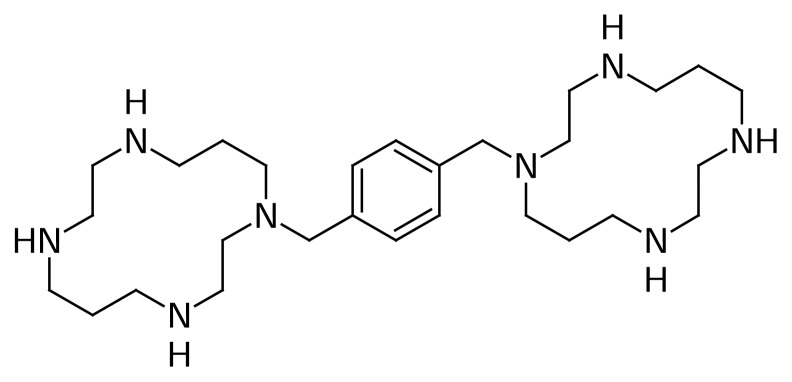
Chemical structure of AMD3100

To image CXCR4 expression in tumors, we utilized the ability of the cyclam function of the prototype CXCR4 inhibitor, AMD3100 to form a complex with ^67^Ga to develop [^67^Ga]-AMD3100 as a possible imaging agent for SPECT. Here we present a detailed preparation and *in vivo* evaluation of [^67^Ga]-AMD3100 in wild type and human tumor-bearing rodents (Figure 1).

## Results and Discussion

### Production

Gallium-67, in the form of GaCl_3_, was prepared by 24 MeV proton bombardment of the ^68^Zn target at Cyclone-30 on a regular basis. The target was bombarded with a current intensity of 170 μA and a charge of 1400 μAh. The chemical separation process was based on the no-carrier-added method.

Radiochemical separation was performed by a two-step ion exchange chromatography method with a yield of higher than 95%. Quality control of the product was performed in two steps. Radionuclidic control showed the presence of 93 (40%), 184 (24%), 296 (22%), 378 (7%) keV gamma energies, all originating from ^67^Ga and showed a radionuclidic purity higher than 99% (E.O.S.). The concentrations of zinc (from the target material) and copper (from the target support) were determined using polarography and shown to be below the internationally accepted levels, i.e. 0.1 ppm for Zn and Cu [11, 12].

The radiochemical purity of the Ga cation was checked in two solvents and chromatographic systems, in 10% ammonium acetate:methanol on Whatman paper, the Ga cation migrated to a higher Rf (0.9), which was totally different from this cation behavior on silica paper in the same solvent as described earlier, while any other Ga radiochemical species remained at the origin (not detected).

**Fig. 2:**
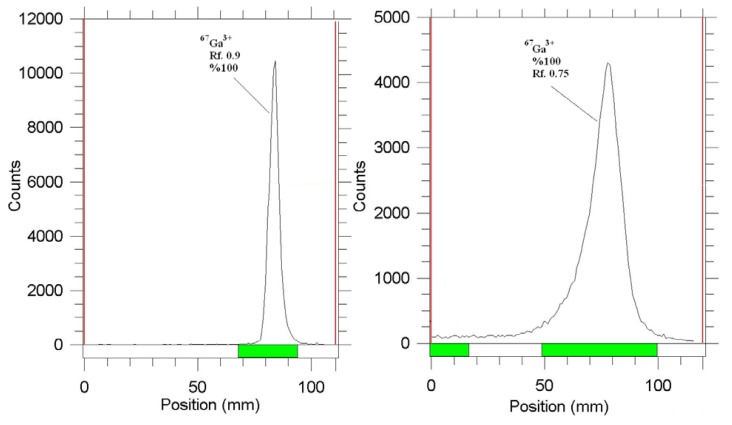
ITLC of [^67^Ga]GaCl_3_ in 10% ammonium acetate:methanol (1:1) mixture on Whatman No.2 papers (left) and 1 M sodium citrate solution on Si papers (right)

Using 1 M sodium citrate solution on Si papers, also, a fast eluting species was observed at the Rf. 0.75, related to Ga-67 citrate species, while any other Ga radiochemical species remained at the origin (not detected). These systems were used also in the detection of the radiolabeled complex (Figure 2).

### Radiolabeling

**Fig. 3:**
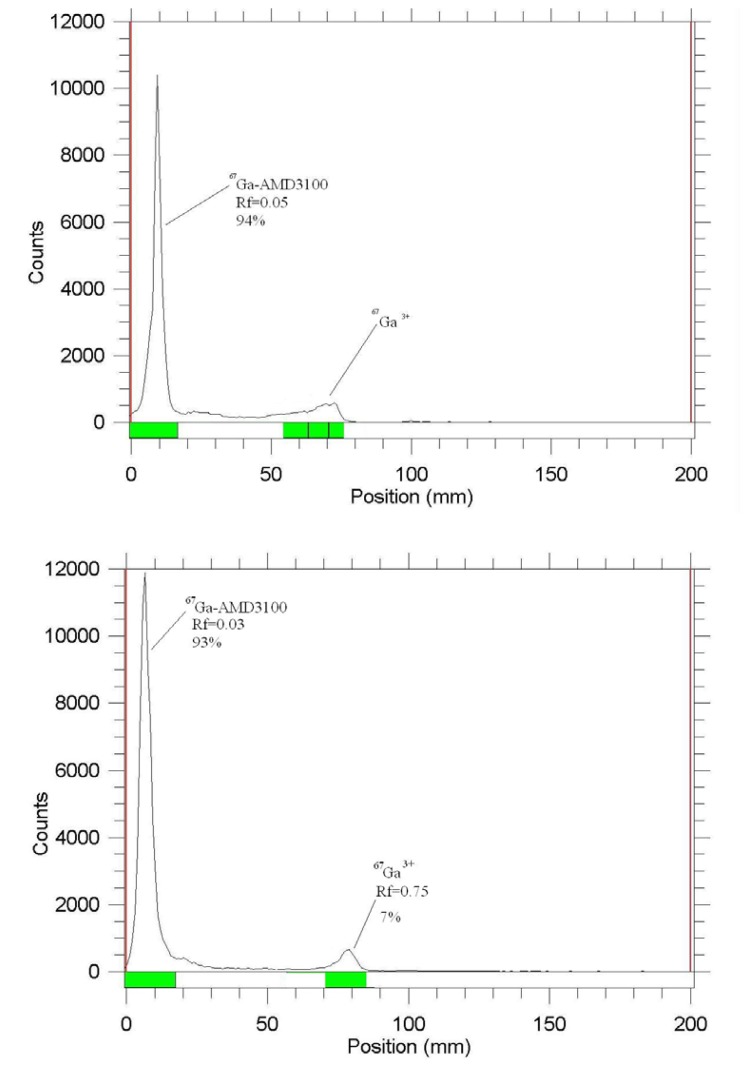
ITLC of [^67^Ga]-AMD3100 in 10% ammonium acetate:methanol (1:1) mixture on Whatman No.2 papers (above) and 1 M sodium citrate solution on Si papers (below)

In the radiolabeling of the most lipophillic complexes, the organic solvent-containing mixtures usually used included, methanol:water, acetonitrile:water mixtures. However, in this case none were able to distinguish ^67^Ga^3+^ from the radiolabeled complex, which might be explained by the fact that the complex itself is a cationic species with significant polarity. Thus, various systems were used in this regard and two very polar systems on polar solid states worked well (Figure 3).

As mentioned earlier, the Ga cation was eluted to the higher R_f_ while the highly polar radiolabeled complex [^67^Ga]-AMD3100, was retained to the origin. It can be assumed that the nature of the radiolabeled complex is polyionic as observed for the Zn(II)AMD3100 complex with four cationic centers.

**Fig. 4:**
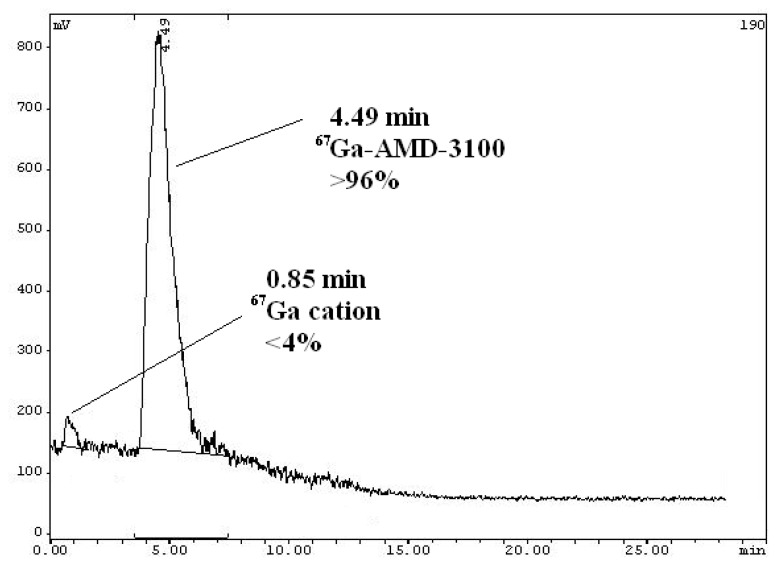
HPLC chromatogram of [^67^Ga]-AMD3100 solution on a reversed-phase column using acetonitrile +0.1% TFA/water + 0.1% TFA,gradient from 10:90 to 90:10.

The cationic nature of the complex was also a major obstacle in the HPLC radioanalysis and a cationic column was preferable. However, we used a reversed-phase column in our settings and it worked with a tolerable difference in the retention times, enough for analytical measurements. The HPLC experiments using acetonitrile/water + 0.1% TFA with a gradient protocol (10:90 to 90:10) was applied. In this system, free Ga eluted at 0.85 minutes while the complex was eluted at 4.49 minutes (scintillation detector) demonstrating a radiochemical purity of higher than 96% using optimized conditions without further purifications (Figure 4).

At room temperature no detectable complex was formed. The best temperature was found to be 55–60 °C. At this temperature, when freshly prepared gallium-67 was used, all the radio-gallium was inserted into the complex. While heating the reaction mixture to over 100 °C or for more than 1 h, the radiochemical yield dropped. The solution was stable at room temperature for up to 4 days post-formulation, allowing good performance of the biological experiments. Before the experiments, the solution passed through a 0.22 μm filter (Millipore).

### Stability

Incubation of [^67^Ga]-AMD3100 in freshly prepared human serum for 2 days at 37 °C showed no loss of ^67^Ga from the complex. The radiochemical purity of the complex remained at 99% for 2 days under physiologic conditions. Table 1 demonstrates the stability data for the complex.

### Biodistribution

**Fig. 5:**
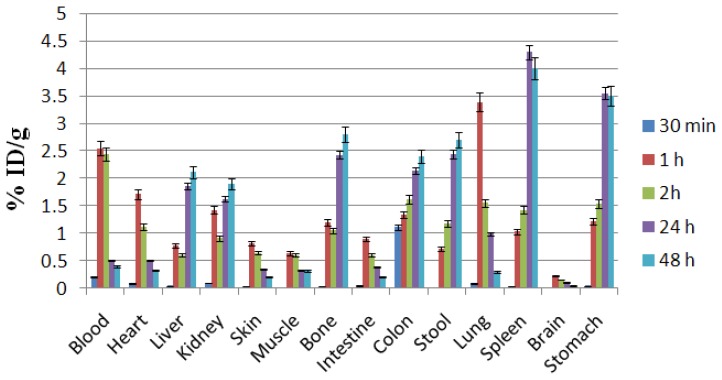
Biodistribution of [^67^Ga]GaCl_3_ (1.85 MBq, 50 μCi) in normal rats 0.5–48 h after IV injection via tail vein (ID/g%: percentage of injected dose per gram of tissue calculated based on the area under curve of 184 keV peak in gamma spectrum) (n=5)

For better comparison, a biodistribution was performed for free Ga^3+^ as well. The ID/g% data are summarized in Fig. 5. At two hours post-injection, the radioactivity was enhanced in the kidneys. This pattern stayed constant up to 24 hours. The radioactivity of the intestines as well as the stomach was high at 2 h which could be due to the metabolism of the radiolabeled complex in the liver. As a bioisoester of the ferric cation, Ga-67 was captured by transferrin in the serum and finally transferred to the liver, which was gradually retained in the liver. The liver activity had also in part been excreted into the hepatobiliary tract in free or other metabolic forms leading to an increase in the activity in the stool and colon. The lungs and spleen are the two reticuloendothelial tissues which also contain transferrin receptors, at which the activity is increased in these organs at some time intervals. Bone uptake was also observed in this case. At the tracer levels, Ga usually does not significantly accumulate in the bones, however being a biohomolog of the ferric cation, areas where Ga-67 normally localizes include: liver (site of highest uptake), bone marrow, spleen, salivary glands, nasopharynx lacrimal glands, breast uptake (especially in pregnant and lactating women), kidneys, and bladder in the first 24 hours.

**Fig. 6:**
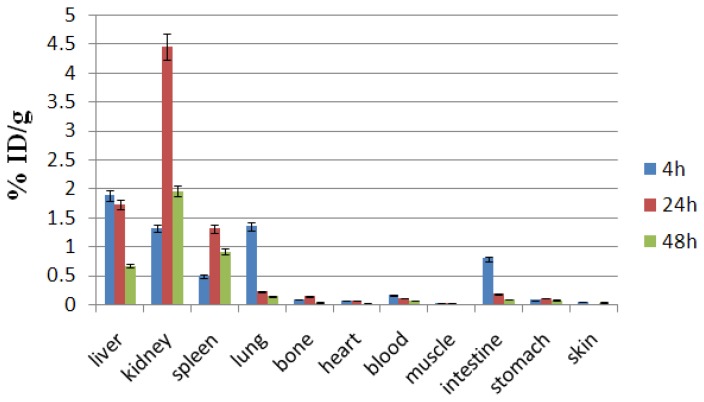
Biodistribution of [^67^Ga]-AMD3100 (1.85 MBq, 50 μCi) in wild rats 4–48 h after IV injection via tail vein (ID/g%: percentage of injected dose per gram of tissue calculated based on the area under curve of 184 keV peak in gamma spectrum) (n=5)

CXCR4 is abundantly expressed in normal tissues such as the lungs, liver, and bone marrow and much less in other tissues [13]. Interestingly, CXCR4 is absent in most healthy tissue cell surfaces and as observed in Figure 6, just the excretion tissues contain the activity. Unlike free cations, [^67^Ga]-AMD3100 is a rapidly washed-out and highly water-soluble complex, majorly excreted through the urinary tract. The high stability of the complex predicted by *in vitro* methods does not allow the detachment of the radio cation into the blood and other organs, thus kidneys are the most important excretion organs and also possibly the critical organs in the dosimetry calculations. Unlike the Ga^3+^ cation, the bones, heart, muscle, skin, and stomach do not demonstrate significant uptakes at all time intervals.

The kidneys, liver, spleen, and lungs are the only significant uptake targets. From the data it can be suggested that [^67^Ga]-AMD3100 is metabolized and/or excreted through the kidneys and hepatic metabolism, respectively. However, no metabolic study was performed to identify the natures of the metabolite(s).

The high kidney uptake can cause an extra dose to surrounding critical tissues including the gonads, which can be an obstacle especially when using therapeutic radionuclides for therapy. However, the decrease in kidney uptake in 48 hours would minimize this effect. In the present study we are using diagnostic radionuclides, at which lower doses are imposed on the organs.

In a single report using ^125^I-anti CXCR4, the spleen has been shown to be a major site of accumulation, possibly due to the presence of CXCR4-containing blood cells. Also, the lungs and liver contain medial receptor sites at their cell surfaces [14]. With respect to this work, the major receptor-rich organ (%ID/g=35 at 24 h post injection), directly or indirectly can be considered the spleen. However, the high kidney uptake is a result of being the major excretion organ due to the high water solubility of the complex, and not the receptor-mediated uptake.

### [^67^Ga]-AMD3100 Imaging in Wild Type Rats

**Fig. 7:**
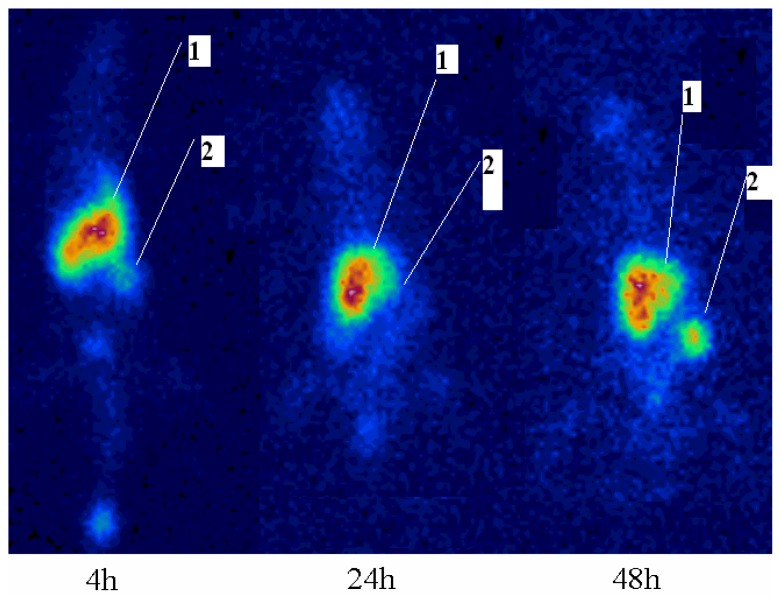
SPECT images of [^67^Ga]-AMD3100 (100 μCi) in wild type rats, 4, 24, and 48 h post injection; 1: abdominal regions (including spleen, liver, and lung); 2: the kidneys

[^67^Ga]-AMD3100 imaging in the wild type rats showed a distinct accumulation of the radiotracer in the CXCR4-containg cells including the spleen, liver, lung, and intestine as described above. Figure 7 demonstrates the abdominal high accumulation related to the mentioned organ uptakes and also due to the polar nature of the complex, where the major excretion site remains the kidneys. The images are similar in pattern to the 4–48 h post-injection.

### [^67^Ga]-AMD3100 Imaging in Human Breast Carcinoma-Bearing Mice

**Fig. 8:**
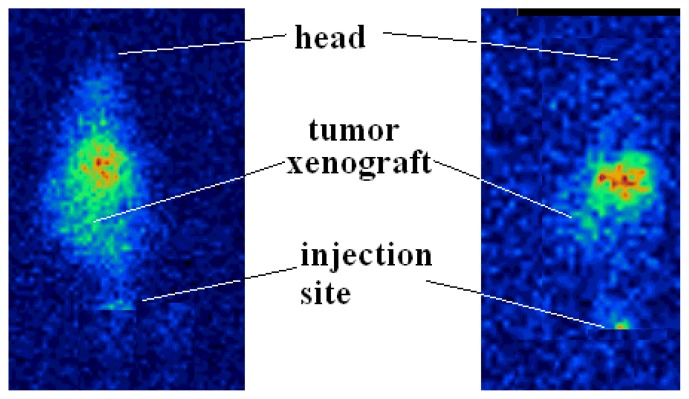
SPECT images of [^67^Ga]-AMD3100 (0.8 MBq, 22 μCi) in human breast carcinoma-bearing bearing mice 4 h (left) and 24 h (right) post-injection

[^67^Ga]-AMD3100 imaging in the human breast carcinoma-bearing mice showed a distinct accumulation of the radiotracer in the tumor tissue 4 and 24 h post-injection (Figure 8). The retention of the radioactive material in the target organ was further investigated by an anatomical study of the tumor position after imaging. Tumor accumulation was not due to Ga^3+^ free cation (like that of Ga-citrate), since gallium scans usually resulted in positive tumor uptake at longer times.

## Experimental

Enriched zinc-68 chloride with a purity of more than 95% was obtained from the Ion Beam Separation Group at Agricultural, Medical, and Industrial Research School (AMIRS). Production of ^67^Ga was performed at the Nuclear Medicine Research Group (AMIRS) 30 MeV cyclotron (Cyclone-30, IBA). AMD3100 hexa hydrochoride was purchased from the Sigma-Aldrich Chemical Co. (Gemany); and the ion-exchange resins from Bio-Rad Laboratories (Canada). Thin layer chromatography (TLC) for cold compounds was performed on polymer-backed silica gel (F 1500/LS 254, 20 × 20 cm, TLC Ready Foil, Schleicher & Schuell^®^, Germany). Normal saline and sodium acetate used for labeling were of high purity and had been filtered through 0.22 μm Cativex filters. Instant thin layer chromatography (ITLC) was performed by counting Whatman No. 2 papers using a thin layer chromatography scanner, Bioscan AR2000, Bioscan Europe Ltd. (France). Analytical high-performance liquid chromatography (HPLC), used to determine the specific activity, was performed by a Shimadzu LC-10AT, armed with two detector systems, a flow scintillation analyzer (Packard-150 TR), and UV-Visible (Shimadzu) using a Whatman Partisphere C-18 column 250×4.6 mm, Whatman, NJ (USA). Analytical HPLC was also used to determine the specific radioactivity of the labeled compound. A standard curve was generated to calculate the mass of the final solution. Biodistribution data were acquired by counting normal saline-washed tissues after weighting on a Canberra™ high purity germanium (HPGe) detector (model GC1020-7500SL). Radionuclidic purity was checked with the same detector.

For the activity measurement of the samples a CRC Capintec Radiometer (NJ, USA) was used. All calculations and ITLC counting were based on the 184 keV peak. Animal studies were performed in accordance with the United Kingdom Biological Council’s Guidelines on the Use of Living Animals in Scientific Investigations, 2nd Ed.

### Production of ^67^Ga

^68^Zn(p,2n)^67^Ga was used as the best nuclear reaction for the production of ^67^Ga. Other impurities could be removed in the radiochemical separation process. After the target bombardment process, chemical separation was carried out in no-carrier-added form. The irradiated target was dissolved in 10 M HCl (15 ml) and the solution was passed through a cation exchange resin (AG 50 W, H^+^ form, mesh 200–400, h:10 cm, Ø:1.3 cm) which had been preconditioned by passing 25 ml of 9 M HCl. The column was then washed by 25 ml of 9 M HCl at a rate of 1 ml/min to remove the copper and zinc ions. To the eluent, 30 ml water plus about 100 ml of a 6 M HCl solution was added. The latter solution was loaded on another exchange resin (AG1X8 Cl^−^ form, 100–200 mesh, h: 25 cm, Ø:1.7 cm) pretreated with 6 M HCl (100 ml). Finally, the gallium-67 was eluted as [^67^Ga]GaCl_3_ using 2 M HCl (50 ml); the whole process took about 60 min.

### Quality Control of the Product

#### Control of radionuclide purity

Gamma spectroscopy of the final sample was carried out by counting in an HPGe detector coupled to a Canberra™ multi-channel analyzer for 1000 seconds.

#### Chemical purity control

This step was carried out to ensure that the amounts of zinc and copper ions resulting from the target material and backing in the final product were acceptable regarding internationally accepted limits. The chemical purity was checked by differential-pulsed anodic stripping polarography. The detection limit of our system was 0.1 ppm for both zinc and copper ions.

### Preparation of [^67^Ga]-AMD3100

The acidic solution (2 ml) of [^67^Ga]GaCl_3_ (111 MBq, 3 mCi) was transferred to a 5 ml-borosilicate vial and heated to dryness using a flow of N_2_ gas at 50–60 °C followed by the addition of an acetate buffer (500 μL, pH 5). Fifty microlitres of AMD3100 hexa hydrochloride dissolved in acetate buffer pH=5 (5 mg/ml ≈63 nmoles) was added to the gallium-containing vial and vortexed at 25, 50, and 80 °C separately. The active solution was checked for radiochemical purity by ITLC and HPLC methods at 0.5, 1, 2, and 4 h after labeling. The final solution of the radiolabeled compounds was then passed through a 0.22 μm filter and the pH was adjusted to 5.5–7.

### Quality Control of [^67^Ga]-AMD3100

#### Radio thin layer chromatography

A 5 μl sample of the final fraction was spotted on the chromatography silica gel plates using a 0.1 M sodium citrate solution as the eluent, Whatman No. 2 paper, and a 10% ammonium acetate:methanol (1:1) mixture as the mobile phase.

#### High-performance liquid chromatography

HPLC was performed with a flow rate of 1 ml/min, pressure: 130 kgF/cm^2^ for 20 min. The radiolabeled compound was eluted using a mixture of two solutions (A: acetonitrile + 0.1%TFA/water + 0.1% TFA, 90:10) using a reversed-phase column Whatman Partisphere C_18_ 4.6 × 250 mm.

### Stability Tests

The stability of the complex was checked according to the conventional ITLC method [15]. A sample of [^67^Ga]-AMD3100 (37 MBq) was kept at room temperature for 4 days while being checked by ITLC at time intervals in order to check the stability in the final product using the above chromatography system. For the serum stability studies, to 36.1 MBq (976 μCi) of [^67^Ga]-AMD3100 was added 500μl of freshly prepared human serum and the resulting mixture was incubated at 37 °C for 4 d, aliquots (5 μl) were analyzed by ITLC.

### Development of Breast Carcinoma-Xenograft Mice for SPECT Studies

Breast carcinoma cells were procured from the American Type Culture Collection (Manassas, VA) and maintained in Leibovitz medium and Dulbecco’s modified Eagle medium, respectively, supplemented with 10% FBS and 100 U/ml of penicillin and streptomycin under standard culture conditions. The cells were cultured at a density of 5000 cells/cm^2^ in 60 mm dishes and were treated the following day with DMSO for 24 to 72 hours, and both adherent and non-adherent cells were collected by brief trypsinization followed by centrifugation. The cells were stained with Trypan blue and counted as live (unstained) and dead (blue-colored) cells using a hemocytometer under a light microscope. The tumor was established by the subcutaneous implantation of exponentially growing 5×10^6^ human breast carcinoma cells mixed with Matrigel (1:1) in the right side of the abdominal region of inbred female BALB/c mice (16–25 g, 6–8 weeks old, Pasteur Institute, Tehran, Iran). The biodistribution studies were performed when the tumor volume reached 7–8 mm^3^. All the animal experiments were approved by the Animal Care Committee of Nuclear Science and Technology Research Institute (NSTRI).

### Biodistribution of [^67^Ga]-AMD3100 in Wild Type Rats and Breast Carcinoma-Xenograft-Bearing Mice

To determine the biodistribution, [^67^Ga]-AMD3100 and ^67^GaCl_3_ were administered to normal rats separately. Also, [^67^Ga]-AMD3100 was administered to tumor-bearing mice and studied for 24–72 h. A volume (50–100 μl) of final radioactive solution containing 1.85±0.2 MBq radioactivity was injected intravenously in each rodent through their tail vein. The total amount of radioactivity injected into each rat was measured by counting the 1 ml syringe before and after injection in a dose calibrator with a fixed geometry. The animals were killed by CO_2_ asphyxiation (after anesthesia induction using a propofol/xylazine mixture) at selected times after injection at the exact time intervals, and the specific activities of different organs were calculated. Dissection began by drawing blood from the aorta, followed by collecting the heart, spleen, kidney, liver, intestine, stomach, lung, bones, muscle, and skin samples. The samples were weighed and their specific activities were determined with an HPGe detector counting the area under the curve of the 184 keV peak. The tissue uptakes were calculated as the percent of the area under the curve of the related photo peak per gram of tissue (% ID/g).

### SPECT Imaging of [^67^Ga]-AMD3100 in Tumor-Bearing Mice and Wild Type Rats

Human breast carcinoma-bearing mice were used for tumor imaging when the tumors reached a size of 0.2–0.4 g, 14–16 weeks after its induction. Images were taken 4 and 24 hours after administration of the tracer by a dual-head SPECT system. The mouse-to-high energy septa distance was 12 cm. The useful field of view (UFOV) was 540 mm×400 mm. The spatial resolution was 10 mm FWHM at the CFOV. Sixty-four projections were acquired for 30 seconds per view with a 64×64 matrix. Also, wild type rats went through imaging 4, 24, and 48 h post-injection.

## Conclusion

The total labeling and formulation of [^67^Ga]-AMD3100 took about 120 minutes, with radiochemical purity >99% (HPLC) and a specific activity: 1800–2000 TBq/mmol. The complex was stable in the presence of human serum (37 °C) and in final formulation for 4 days. The biodistribution of the labeled compound in the vital organs of wild type Sprague-Dawley rats demonstrated a significant uptake in the liver, spleen, and lungs which have been shown to be major expression sites for CXCR4 in rats. [^67^Ga]-AMD3100 is metabolized 65% and/or excreted through the kidneys and metabolized 35% by the liver. The initial SPECT images and biodistribution results in the wild type rats matched each other and demonstrated rapid washout of the tracer from the urinary tract. The SPECT images in human breast carcinoma-bearing mice demonstrated a detectable tumor uptake in 48 h post-injection. Considering the spleen as the target organ, the best target:non target ratios were obtained 48 h post-injection (spleen:blood ratio; 14.5 and spleen:muscle ratio ; 88.4). It is suggested that [^67^Ga]-AMD3100 could be a possible SPECT tracer.

## Figures and Tables

**Tab. 1:** The stability of the radiolabeled complex in the presence of human serum at 37 °C and final formulation in 48 hours using RTLC and 1 M sodium citrate solution on Si paper

Stability/time	4(h)	8(h)	12(h)	24(h)	48(h)
Final preparation	93±1%	92±0.5%	91±0.8%	90±1%	88±1%
Human serum	93±1%	90±1%	90±2%	88±1%	86±1%
